# Blood bacterial DNA signatures in a prospective cohort of patients with MASLD cirrhosis

**DOI:** 10.1097/HC9.0000000000000722

**Published:** 2025-06-09

**Authors:** Suet-Ying Kwan, Lillian I. Dolapchiev, Caren I. Sanchez, Tiffany L. Calderone, Jessica I. Sanchez, Megha B. Bhongade, Ahmed El Sabagh, Darrel W. Cleere, Nakul Gupta, Prasun K. Jalal, David W. Victor, Laura Beretta

**Affiliations:** 1 Department of Molecular and Cellular Oncology, The University of Texas MD Anderson Cancer Center, Houston, Texas, USA; 2 Margaret M. and Albert B. Alkek Department of Medicine, Section of Gastroenterology and Hepatology, Baylor College of Medicine, Houston, Texas, USA; 3 Department of Gastroenterology, Houston Methodist Hospital, Houston, Texas, USA; 4 Department of Radiology, Houston Methodist Hospital, Houston, Texas, USA

**Keywords:** bacteria, biomarkers, cirrhosis, HCC, MASLD

## Abstract

**Background:**

Predictive biomarkers are needed to identify individuals with metabolic dysfunction–associated steatotic liver disease (MASLD), at high risk for HCC. Our study aimed to determine whether the detection of circulating bacterial DNA could be associated with HCC development in MASLD patients with liver cirrhosis.

**Methods:**

We developed a multicenter prospective cohort of patients with cirrhosis undergoing surveillance for HCC by contrast-enhanced magnetic resonance imaging. In a nested cohort study, we performed 16S rRNA sequencing of cell-free DNA extracted from 343 longitudinal plasma samples collected from 151 MASLD patients with cirrhosis. Among the 151 patients, 25 developed HCC during follow-up.

**Results:**

Following in silico decontamination approaches, variations in circulating bacterial DNA profiles were significantly associated with HCC development, cirrhosis severity, and male gender. Many of the identified taxa were well-known human-associated bacteria. Patients who developed HCC during follow-up showed an enrichment in *Tsukamurella* and *Bacteroides*, and depletion of *Natronomonas*. Associations with HCC development remained for the identified bacteria, after adjusting for cirrhosis severity and male gender. Acidobacteriota, *Tsukamurella*, and *Staphylococcaceae* showed markedly improved prediction of HCC within 12 months prior to diagnosis [Acidobacteriota: AOR=2.87 (1.36–6.04), *p*=0.006; *Tsukamurella*: AOR=2.80 (1.34–5.85), *p*=0.006; *Staphylococcaceae*: AOR=2.52 (1.19–5.36), *p*=0.016]. Circulating bacterial DNA profiles associated with male gender and cirrhosis severity were different from those observed for HCC and showed considerable overlap in significant taxa. These included enrichment of the lineage Gammaproteobacteria, Enterobacterales, and *Rheinheimera*.

**Conclusions:**

The identified circulating bacterial DNA signatures may have utility in personalized approaches to HCC surveillance in MASLD patients.

## INTRODUCTION

Liver cancer is a major contributor to cancer deaths worldwide, and the number of liver cancer cases is expected to increase by 55% in the next 2 decades.^1^ In the United States, while the overall mortality rate of HCC has begun to decrease, HCC incidence is still increasing in several states.^2^,^3^ HCC prognosis remains poor despite recent efforts to detect localized HCC.^2^ Suboptimal screening and risk stratification methods have limited the effectiveness of HCC surveillance in detecting early tumors.^4^,^5^ HCC surveillance can be improved using novel biomarkers to stratify patients into low-risk and high-risk groups. These biomarkers need to be evaluated in prospective cohorts of at-risk subjects under surveillance for HCC, with imaging allowing for the detection of small early-stage lesions. To that end, we developed a multicenter prospective cohort of patients with cirrhosis undergoing surveillance for HCC with contrast-enhanced MRI.

Individual factors such as etiology, age, and gender must be incorporated into personalized approaches to surveillance. In particular, risk prediction models are needed to identify those affected by metabolic dysfunction–associated steatotic liver disease (MASLD) who are at risk of developing HCC.^6^ MASLD impacts about one-third of adults worldwide.^7^ MASLD can progress to steatohepatitis, cirrhosis, or HCC, and MASLD-related HCC cases are expected to increase due to factors such as diabetes and the rise in obesity.^8^,^9^ We and others have shown that the gut microbiome plays an important role in the progression of MASLD,^10^,^11^,^12^,^13^ liver fibrosis,^14^,^15^,^16^,^17^,^18^ and HCC.^19^,^20^ We and others have also proposed that the gut microbiome could serve as a noninvasive biomarker for early detection of HCC.^21^,^22^,^23^ HCC patients have a microbiome signature distinct from individuals without HCC, and studies suggest the gut microbiome and its metabolites can affect processes associated with HCC, such as immune responses and metabolism.^24^ Using a global metabolomics approach on our prospective cohort of patients with cirrhosis, we identified several microbiota-derived metabolites in serum as main biomarkers of HCC risk within a year prior to HCC diagnosis.^25^ The use of blood bacterial DNA signatures to predict HCC outcome may therefore be a potential complementary strategy to metabolomics. Human blood contains a low biomass of circulating microbial cell-free DNA (cfDNA), originating predominantly from bacteria.^26^ In healthy individuals, there is no evidence for a core blood microbiome, suggesting a transient and sporadic translocation of commensal microbes into the blood from other areas of the body.^27^ However, studies have found common bacterial taxa such as *Bacteroides*, *Escherichia*/*Shigella*, and *Staphylococcus* in conditions such as cirrhosis or inflammatory diseases.^28^ There is also evidence that genetic materials originating from the microbiome can be found in the blood cfDNA of cancer patients and could be exploited for patient stratification.^29^ Distinct circulating plasma microbiome profiles were previously identified in patients with liver cirrhosis or HCC.^30^,^31^,^32^ The origin of circulating bacterial cfDNA is unknown, as studies have identified members of various microbiome niches, including the gut, oral cavity, airways, and skin.^26^ To explore the utility of circulating microbial markers in predicting risk of HCC, we applied in this study, 16S rRNA sequencing of cfDNA to a large number of longitudinal samples collected in our prospective surveillance cohort, from patients with MASLD-associated cirrhosis who developed or did not develop HCC during surveillance.

## METHODS

### Patient cohort

This study was conducted in accordance with the Declaration of Helsinki and approved by the Institutional Review Board of all participating institutions. At recruitment, written informed consent was obtained from each participant. Medical history was collected to confirm eligibility (above 18 y of age, confirmed diagnosis of cirrhosis, no clinical evidence of significant hepatic decompensation). Cirrhosis was diagnosed using composite clinical, biochemical, hematological, imaging, or histological criteria. Date of birth, gender, race, ethnicity, liver cirrhosis etiology, ascites status, encephalopathy status, Child–Pugh score, Child–Pugh class, MELD score, year of cirrhosis diagnosis, diabetes, and date of diabetes onset were collected. A nested group of 151 patients under surveillance for HCC with contrast-enhanced MRI was selected from a large multicenter prospective cohort initiated in 2017 (Supplemental Table S1, http://links.lww.com/HC9/C3). The patients for this nested cohort study were recruited at Houston Methodist Hospital (n=72) and Baylor College of Medicine (n=79). All participants were selected for having MASLD-associated cirrhosis and were followed for the duration of the study with contrast-enhanced MRI and blood collection, at time intervals defined by their standard of care surveillance at participating institutions. The average duration of follow-up was 31 months (up to 90.1 mo). At each imaging, liver lesions (if any) were classified by the Liver Imaging Reporting and Data System (LI-RADS) classification system. Among the 151 patients, 25 developed HCC during follow-up.

### Cell-free DNA (cfDNA) extraction

At enrollment and at each follow-up visit, blood samples were collected in purple top EDTA vacutainers and centrifuged at 4 °C for 10 minutes at 900*g*. Plasma supernatants were transferred to 2 mL microcentrifuge tubes and then centrifuged for 10 minutes at 20,000*g* at 4 °C to remove cellular components. After centrifugation, supernatants were aliquoted into cryovials and stored at −80 °C until cfDNA extraction. cfDNA was extracted from 1 mL of each of the 368 plasma samples in 18 batches, using the QIAamp Circulating Nucleic Acid Kit (catalog #55114, Qiagen) with the QIAvac 24 Plus vacuum manifold (catalog #19413, Qiagen). For each batch, a negative extraction control was also prepared using only the kit extraction reagents. To minimize DNA contamination, DNA extraction was performed in a flow hood. The work surface was exposed to 254 nm UV light within 10 cm for 30 minutes prior to extraction. DNA AWAY was used to decontaminate surfaces and tools, and dedicated DNase-free and nucleic acid-free tubes were used. The empty water bath was wiped down with DNA AWAY before being filled with autoclaved distilled water.

### 16S rRNA sequencing

16S rRNA sequencing was performed at the Sequencing and Analysis Core of the Baylor College of Medicine Alkek Center for Metagenomics and Microbiome Research, using 8.5 µL of extracted cfDNA from plasma samples and extraction controls. Sequencing methods were adapted from those developed for the NIH-Human Microbiome Project and the Earth Microbiome Project. The V4 region of the bacterial 16S rRNA gene was amplified by PCR (515F forward primer: 5′-GTGCCAGCMGCCGCGGTAA; 806R reverse primer: 5′-GGACTACHVGGGTWTCTAAT-3′) and sequenced on the Illumina MiSeq platform by paired-end sequencing. Primers used for amplification contained adapters for Illumina platform sequencing. Additionally, single-index barcodes were incorporated into the reverse primer, allowing direct pooling and sequencing of PCR products. Demultiplexed read pairs underwent an initial quality filtering using bbduk.sh (BBMap, version 38.82), removing Illumina adapters, PhiX reads, and reads with a Phred quality score <15 and length <100 bp after trimming. Quality-controlled reads were then merged using bbmerge.sh and further filtered via VSEARCH. Sequences were clustered into operational taxonomic units (OTUs) at a similarity cutoff of 97% using the UPARSE algorithm. OTUs were subjected to taxonomy assignment by mapping to the SILVA database v138.

Prior to any analysis, unmapped OTUs and taxa detected in <10% of all patients were removed. Extraction batch effects were also assessed using principal coordinates analysis (PCoA) of the weighted UniFrac distances, calculated from OTU abundances (Supplemental Figure S1A, http://links.lww.com/HC9/C4). As batch 18 was a strong outlier, all samples from this batch were excluded from downstream analyses. Two additional outliers were further excluded based on low-sequencing depth (<2000 reads) or rarefaction curves (Supplemental Figure S1B, http://links.lww.com/HC9/C4), leaving for downstream analyses, a total of 343 samples from the 151 patients, with an average of 2.3 samples per patient (range 1–13), and 17 extraction controls. The final 343 samples had an average read depth of 12,624 reads (range=2253–34,956).

### Statistical analyses

Statistical analyses were performed in R (version 4.4.1; R Foundation for Statistical Computing). PCoA was performed using the “cmdscale” function and weighted UniFrac distances of OTU abundance. Using the “capscale” function of the Vegan package and weighted UniFrac distances of OTU abundance, distance-based redundancy analysis (dbRDA) was performed to determine the influence of demographic and clinical parameters on circulating bacterial profiles. To determine the significance of the model and of each explanatory variable, ANOVA-like permutation and marginal tests were performed using the “anova.cca” function of the Vegan package. Differences in bacterial abundances between groups were assessed with Analysis of Compositions of Microbiomes with Bias Correction 2 (ANCOM-BC2)^33^ and taxonomic count data, using the “ancombc2” function of the ANCOMBC package. Taxa with greater abundance in the negative extraction controls than in the biological samples were excluded from differential analyses. Following differential abundance analysis, ANCOM-BC2 handles excess zeros via a sensitivity analysis that assesses the robustness of results to the choice of pseudocount.^33^ Taxa with a Benjamini–Hochberg-adjusted *q*-value (*q*) <0.2, and passing sensitivity analysis, were considered significantly different between comparison groups. For HCC development and gender, primary ANCOM-BC2 analysis was performed. For Child–Pugh class (consisting of classes A, B, and C), the pattern test was performed to test for monotonically increasing and decreasing patterns across the 3 ordinal groups. ANCOM-BC2 was repeated for data with in silico decontamination by Source tracking for Contamination Removal in microBiomes (SCRuB).^34^ Receiver operating characteristic (ROC) curve analysis using taxonomic count data was additionally performed using the pROC and ROCR packages to determine the ability of individual and combined taxa to discriminate between patient groups. Using male gender, Child–Pugh class C or HCC development as the binary outcome, fitted probabilities were used for graphing ROC curves and computing the AUC. Binary logistic regression was additionally performed for individual taxa, using male gender, Child–Pugh class C or HCC development as the binary outcome. Adjusted ORs adjusted odds ratio (AORs) and 95% CIs were calculated for samples with a high abundance of the bacterial taxon.

## RESULTS

### Nested cohort study of MASLD patients with cirrhosis under HCC surveillance by contrast-enhanced MRI

We used a nested cohort of 151 MASLD patients with cirrhosis, from a multicenter prospective cohort of patients with cirrhosis under surveillance for HCC by contrast-enhanced MRI. The demographic and clinical parameters of the study participants at enrollment are shown in Supplemental Table S1, http://links.lww.com/HC9/C3. Most participants were female (58.3%) and non-Hispanic White (72.8%). At recruitment, the median age was 63, the median BMI was 33.8, and 68.9% of all participants had diabetes. The presence of alcohol co-etiology was found in 9.9% of the participants. Most patients had Child–Pugh class B (CP-B, 49%), followed by class A (CP-A, 37.7%) and class C (CP-C, 13.2%). Among the 151 patients, 25 developed HCC during follow-up.

Clinical data, imaging, and biospecimens were collected between August 2017 and February 2024. Patient outcome was again reviewed in September 2024. Following the removal of extraction and sequencing outliers, a total of 343 plasma samples from the 151 patients were included in the analysis. These included 281 plasma samples collected from 126 patients who never developed HCC, with an average of 2.2 samples per patient (range 1–12). The remaining 62 plasma samples were collected from the 25 patients who developed HCC during follow-up, with an average of 2.5 samples per patient (range 1–13). These 62 plasma samples were collected at a median of 11.5 months prior to HCC diagnosis (up to 71.5 mo).

### Bacterial DNA profiles in the cfDNA of MASLD patients with cirrhosis

All 343 plasma samples from the 151 patients with cirrhosis and 17 negative extraction batch controls were subjected to cfDNA extraction and 16S rRNA sequencing. A total of 842 taxa were detected in at least 10% of the 151 patients with cirrhosis. As anticipated, alpha diversity measures of richness (Chao1) and richness and evenness (Shannon index) were significantly lower in extraction batch controls than in plasma samples (Supplemental Figure S2, http://links.lww.com/HC9/C4). Prior to any downstream analysis, potential contaminants were removed by excluding 243 taxa with read counts in extraction controls equal to or greater than read counts in plasma samples. The remaining 599 taxa comprised 25 phyla, 47 classes, 101 orders, 170 families, and 256 genera (Supplemental Table S2, http://links.lww.com/HC9/C5). Among the 256 genera, 10 (3.9%) were found in all plasma samples, while 122 (47.6%) were detected in <25% of all plasma samples. A median of 90 genera (range 58–127) were detected per plasma sample. Bacteroidota and Proteobacteria were the most dominant phyla (5432.0 and 3377.4 reads, respectively). Within the Bacteroidota phylum, the large majority of reads were from the *Flavobacteriaceae* family (4563.3 reads). Within the Proteobacteria phylum, the most abundant families were *Comamonadaceae*, *Burkholderiaceae*, *Moraxellaceae*, *Alteromonadaceae*, and *Pseudomonadaceae* (680.7, 622.6, 579.2, 368.6, and 219.0 reads, respectively).

### Demographic and clinical parameters affecting blood bacterial DNA profiles

**FIGURE 1:**
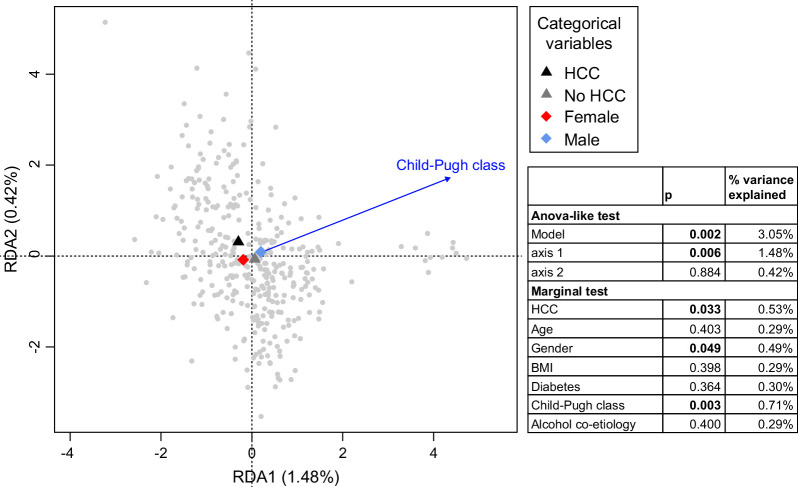
Relationship between demographic and clinical parameters, and plasma bacterial taxa. dbRDA was conducted to determine the relationship between selected parameters and plasma bacterial profiles. The variables with significant effects are shown together with an ANOVA-like significance test of the model. Abbreviations: BMI, body mass index; dbRDA, distance-based redundancy analysis; RDA, redundancy analysis.

We evaluated whether clinical and demographic parameters were associated with inter-sample variation in plasma bacterial DNA profiles. In a distance-based RDA analysis, bacterial profiles were used as the response variable, while age, gender, alcohol co-etiology, Child–Pugh class, BMI, diabetes, and HCC outcome were used as explanatory variables (Figure 1). The model was statistically significant (*p*=0.002, 3.05% variation explained) with the main contribution to variation observed for Child–Pugh class (*p*=0.003, 0.71% variation explained), HCC development (*p*=0.033, 0.53% variation explained), and gender (*p*=0.049, 0.49% variation explained).

### Circulating bacterial DNA signatures associated with the development of HCC

**FIGURE 2:**
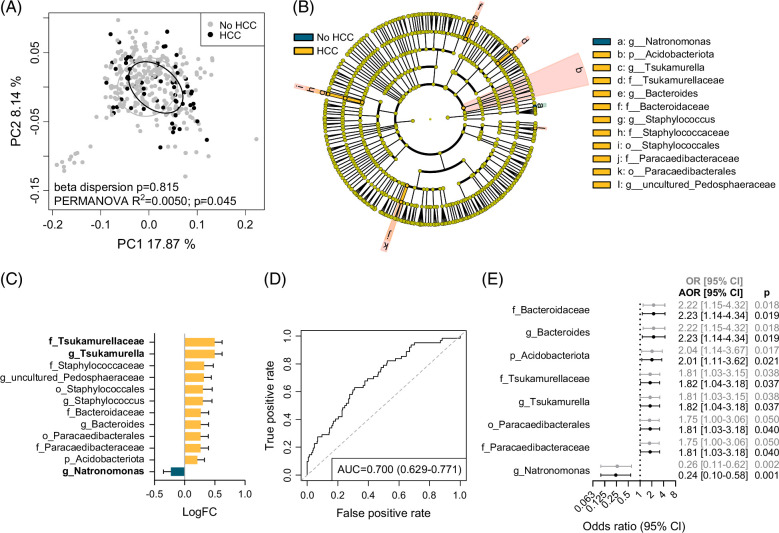
Circulating bacterial taxa with altered abundance in patients with cirrhosis who developed HCC versus those that did not. (A) Beta diversity of plasma bacterial profiles in patients with cirrhosis by HCC development. The PCoA plot is based on weighted UniFrac distances, with samples grouped by HCC development. (B) Cladogram showing taxa with significantly different bacterial abundance between an HCC outcome and no HCC outcome (ANCOM-BC2 *q*<0.2 and AUC ≥0.55). (C) Log fold changes of taxa from (B). Taxa that remained significant by ANCOM-BC2 after in silico decontamination are in bold. (D) ROC curve showing the AUC (95% CI) for taxa from (B), in classifying patients with cirrhosis that developed HCC versus those that did not. (E) Forest plot of taxa from (B), which were also significant by binary logistic regression, after adjusting for gender and Child–Pugh class. ORs and AORs (95% CI) for HCC development are shown for high bacterial abundance. Abbreviations: ANCOM-BC2, Analysis of Compositions of Microbiomes with Bias Correction 2; AORs, adjusted odds ratios; ORs, odds ratios; PCoA, principal component analysis; ROC, receiver operating characteristic.

PCoA analysis confirmed that plasma bacterial DNA profiles were significantly distinct between those that developed HCC versus those that did not (beta dispersion *p*=0.815, PERMANOVA *p*=0.045) (Figure 2A). To identify bacteria enriched or depleted in patients who developed HCC, differential analysis was performed using ANCOM-BC2 on taxa from the phylum to genus level. ROC curve analysis was additionally performed to assess the discriminative power of each individual taxon. Twelve taxa were identified as significant, based on an ANCOM-BC2 *q*<0.2 and area under the ROC curve (AUC) ≥0.55. (Figures 2B, C). Taxa with a significant positive association with HCC development included the *Tsukamurellaceae* family/*Tsukamurella* genus (log FC=0.50, *q*=0.001, AUC=0.588), uncultured *Pedosphaeraceae* (log FC=0.32, *q*=0.040, AUC=0.551), Staphylococcales/*Staphylococcaceae* family/*Staphylococcus* genus (log FC=0.31, *q*=0.121, AUC=0.568), Paracaedibacterales/*Paracaedibacteraceae* (log FC=0.27, *q*=0.111, AUC=0.566); *Bacteroidaceae* family/*Bacteroides* genus (log FC=0.27, *q*=0.118, AUC=0.563), and Acidobacteriota (log FC=0.21, *q*=0.163, AUC=0.562). Conversely, the only taxon with a significant negative association with HCC development was *Natronomonas* (log FC=−0.23, *q*=0.166, AUC=0.633). Among these 12 taxa, 3 remained significant after in silico decontamination of biological samples using negative extraction controls (Figure 2C): *Tsukamurellaceae*/*Tsukamurella* (log FC=0.26, *q*=0.198) and *Natronomonas* (log FC=−0.48, *q*=0.008). When combined, the non-redundant 9 taxa discriminated between HCC development or not, with AUC=0.700 (0.629–0.771) (Figure 2D). Logistic regression analysis was performed for each of the 12 taxa, with 8 of them displaying a significant association with HCC development, after adjusting for gender and Child–Pugh class (Figure 2E). The strongest associations were observed for *Bacteroidaceae*/*Bacteroides* [AOR=2.23 (1.14–4.34), *p*=0.019], Acidobacteriota [AOR=2.01 (1.11–3.62), *p*=0.021], and *Tsukamurellaceae*/*Tsukamurella* [AOR=1.82 (1.04–3.18), *p*=0.037]. A negative association with HCC outcome was observed for *Natronomonas* [AOR=0.24 (0.10–0.58), *p*=0.001].

**FIGURE 3:**
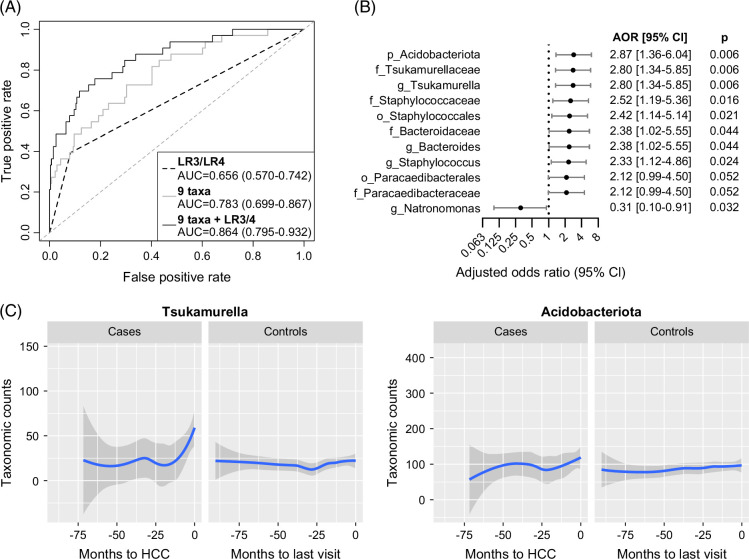
Performance of HCC-associated taxa in predicting risk for HCC within 12 months prior to HCC diagnosis. (A) ROC curves showing the AUC (95% CI) for non-redundant HCC-associated taxa from Figure 2B, for the presence of LI-RAD 3/4 lesions, and for their combination, in classifying patients with cirrhosis that developed HCC within 12 months versus those that did not develop HCC. (B) Forest plot of HCC-associated taxa from Figure 2B, which were also significant by binary logistic regression, after adjusting for gender and Child–Pugh class. ORs and AORs (95% CI) for HCC development within 12 months prior to HCC diagnosis are shown for high bacterial abundance. (C) The overall trend in abundances after in silico decontamination of *Tsukamurella* (left) and Acidobacteriota (right) over time in Cases versus Controls. The blue lines represent locally estimated scatterplot smoothing (loess) regression lines, and the dark gray areas represent the 95% CIs calculated by t-based approximation. Abbreviations: AORs, adjusted odds ratios; LI-RADS, Liver Imaging Reporting and Data System; ROC, receiver operating characteristic.

When including only samples collected within 12 months prior to HCC diagnosis, the HCC-associated taxa showed greater discriminatory power between patients with cirrhosis who developed HCC within 12 months versus those who did not develop HCC [AUC=0.783 (0.699–0.867)] (Figure 3A). In addition, some patients with cirrhosis presented with liver lesions characterized as LI-RADS-3 or LI-RADS-4 lesions, for a total of 40 paired samples. While by itself the presence of such lesions did not predict HCC development [AUC=0.656 (0.570–0.742)], a combination of the presence of these lesions with the 9 unique HCC-associated taxa further improved their performance in predicting HCC within 12 months [AUC=0.864 (0.795–0.932)] (Figure 3A). Furthermore, associations of each taxon with HCC development by logistic regression were mostly stronger when only including samples collected within 12 months prior to HCC diagnosis (Figure 3B). Stronger associations were observed for Acidobacteriota [AOR=2.87 (1.36–6.04), *p*=0.006], *Tsukamurellaceae*/*Tsukamurella* [AOR=2.80 (1.34–5.85), *p*=0.006], and *Bacteroidaceae*/*Bacteroides* [AOR=2.38 (1.02–5.55), *p*=0.044]. Notably, the abundances of *Tsukamurella* and Acidobacteriota after in silico decontamination increased over time in Cases up until the time of HCC diagnosis, while abundance levels remained relatively constant in Controls over time (Figure 3C). While not significant for HCC when including all timepoints, a significant association with HCC within 12 months was observed for Staphylococcales [AOR=2.42 (1.14–5.14), *p*=0.021], the *Staphylcoccaceae* family [AOR=2.52 (1.19–5.36), *p*=0.016], and the *Staphylococcus* genus [AOR=2.33 (1.12–4.86), *p*=0.024].

### Circulating bacterial DNA signatures are associated with male gender and with Child–Pugh class

**FIGURE 4:**
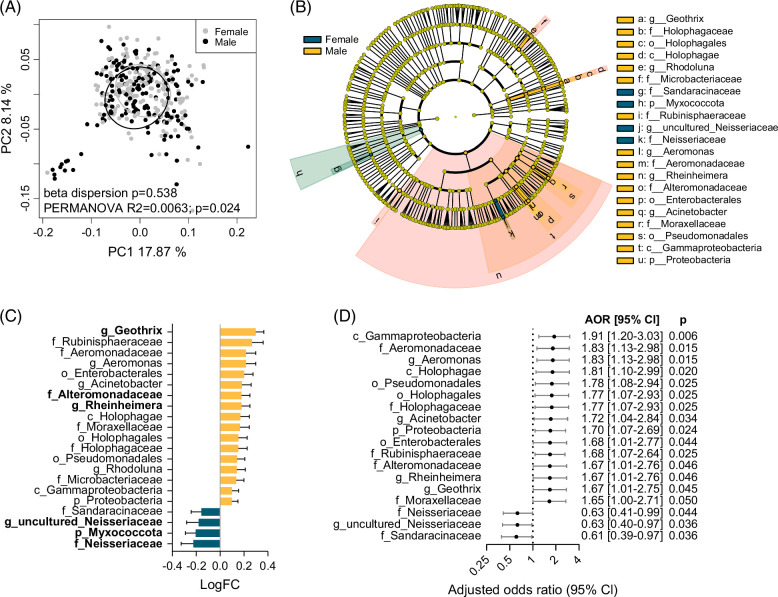
Circulating bacterial taxa with altered abundance in male patients with cirrhosis. (A) Beta diversity of plasma bacterial profiles in patients with cirrhosis by gender. The PCoA plot is based on weighted UniFrac distances, with samples grouped by gender. (B) Cladogram showing taxa with significantly different bacterial abundance between male and female patients (ANCOM-BC2 *q*<0.2 and AUC ≥0.55). (C) Log fold changes of significant taxa from (B) in male versus female patients. The taxa that remained significant by ANCOM-BC2 after in silico decontamination are in bold. (D) Forest plot of significant taxa from (B), which were also significant by binary logistic regression, after adjusting for HCC development and Child–Pugh class. AORs (95% CI) for male gender are shown for high bacterial abundance. Abbreviations: ANCOM-BC2, Analysis of Compositions of Microbiomes with Bias Correction 2; AORs, adjusted odds ratios; Log FC, log fold change; PCoA, principal component analysis.

**FIGURE 5:**
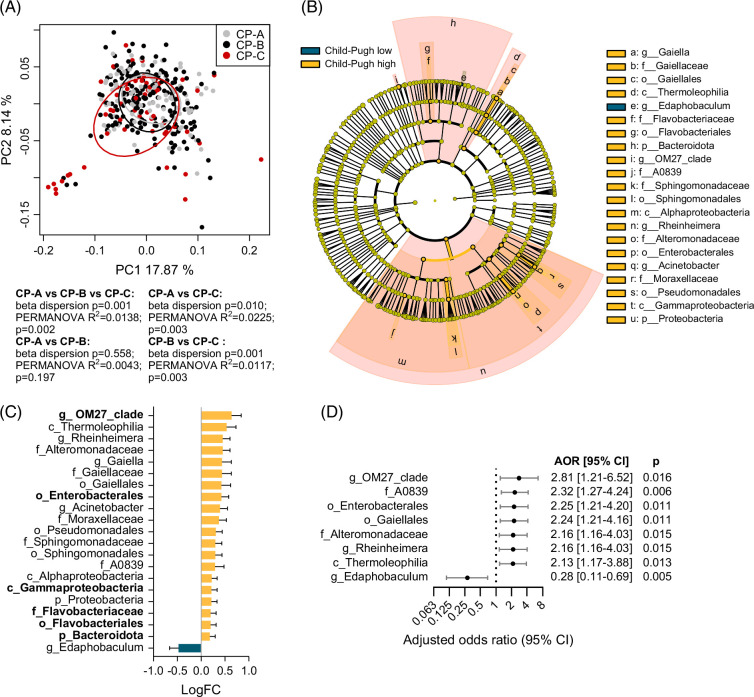
Circulating bacterial taxa with altered abundance across Child–Pugh classes (CP). (A) Beta diversity of plasma bacterial profiles in patients with cirrhosis by Child–Pugh class (CP). The PCoA plot is based on weighted UniFrac distances, with samples grouped by CP class. (B) Cladogram showing taxa with significantly different bacterial abundance across CP classes (ANCOM-BC2 pattern test *q*<0.2 and AUC ≥0.55). (C) Log fold changes of taxa from (B) for CP-C versus CP-A. The taxa that remained significant by ANCOM-BC2 after in silico decontamination, for CP-C versus CP-A, are in bold. (D) Forest plot of taxa from (B), which were also significant by binary logistic regression, after adjusting for HCC development and gender. AORs for CP-C (95% CI) are shown for high bacterial abundance. Abbreviations: ANCOM-BC2, Analysis of Compositions of Microbiomes with Bias Correction 2; AORs, adjusted odds ratios; Log FC, log fold change; PCoA, principal component analysis.

Beta diversity analysis by PCoA plot confirmed that the bacterial DNA profiles were significantly distinct between males and females (beta dispersion *p*=0.538, PERMANOVA *p*=0.024) (Figure 4A). Beta diversity was also significantly different between different Child–Pugh classes, with CP-C being the most distinct class, driving the significant association of Child–Pugh class with bacterial DNA profiles (CP-A vs. CP-B: beta dispersion *p*=0.558, PERMANOVA *p*=0.197; CP-A vs. CP-C: beta dispersion *p*=0.010, PERMANOVA *p*=0.003; CP-B vs. CP-C: beta dispersion *p*=0.001, PERMANOVA *p*=0.003) (Figure 5A). To identify bacterial taxa associated with male gender or with Child–Pugh class, differential and pattern analyses by ANCOM-BC2 were performed on taxa from the phylum to genus level. ROC curve analyses were additionally performed.

A total of 21 taxa were identified as significant between males and females, based on an ANCOM-BC2 *q*<0.2 and AUC ≥0.55 (Figures 4B, C). Taxa with a significant positive association with male gender included Holophagae/Holophagales/*Holophagaceae* family (log FC=0.17, *q*=0.070, AUC=0.554) and *Geothrix* genus (log FC=0.30, *q*<0.001, AUC=0.551). Other taxa enriched in males belonged to Proteobacteria (log FC=0.10, *q*=0.016, AUC=0.561) and Gammaproteobacteria (log FC=0.10, *q*=0.145, AUC=0.564): Enterobacterales (log FC=0.20, *q*=0.023, AUC=0.575); *Aeromonadaceae* family/*Aeromonas* genus (log FC=0.22, *q*=0.027, AUC=0.565); *Alteromonadaceae* family/*Rheinheimera* genus (log FC=0.18, *q*=0.043, AUC=0.568); and Pseudomonadales (log FC=0.14, *q*=0.081, AUC=0.567). In contrast, taxa reduced in male patients included Myxococcota (log FC=−0.21, *q*=0.054, AUC=0.559) and the *Neisseriaceae* family/uncultured *Neisseriaceae* (log FC=−0.22, *q*=0.088, AUC=0.555). Among these 21 taxa, 6 remained significant after in silico decontamination of biological samples using negative extraction controls (Figure 4C). Logistic regression analysis was performed for each of the 21 taxa, with 18 of them displaying a significant association with male gender (*p*<0.05), after adjusting for HCC development and Child–Pugh class (Figure 4D). The strongest associations were observed for Gammaproteobacteria [AOR=1.91 (1.20–3.03), *p*=0.006], *Aeromonadaceae*/*Aeromonas* [AOR=1.83 (1.13–2.98), *p*=0.015], Pseudomonadales [AOR=1.78 (1.08–2.94), *p*=0.025], and Holophagae/Holophagales/*Holophagaceae* family [AORs=1.77–1.81 (1.07–2.99), *p*=0.020–0.025].

A total of 21 taxa were identified as significantly associated with Child–Pugh class, based on an ANCOM-BC2 *q*<0.2 and AUC ≥0.55 (Figures 5B, C). Taxa significantly increasing with Child–Pugh severity included Thermoleophilia (log FC=0.54, *q*=0.032, AUC=0.585), with the majority of reads assigned to the Gaiellales/*Gaiellaceae* family/*Gaiella* genus (log FC=0.43, *q*=0.049, AUC=0.580); Flavobacteriales/*Flavobacteriaceae* (log FC=0.20, *q*=0.193, AUC=0.577); Bacteroidota (log FC=0.19, *q*=0.176, AUC=0.570); and *OM27_clade* (log FC=0.64, *q*=0.032, AUC=0.555). Other taxa enriched with increasing Child–Pugh severity were the *A0839* family (log FC=0.30, *q*=0.193, AUC=0.566); Gammaproteobacteria (log FC=0.22, *q*=0.193, AUC=0.565); Enterobacterales (log FC=0.43, *q*=0.083, AUC=0.585); and *Alteromonadaceae*/*Rheinheimera* (log FC=0.46, *q*=0.032, AUC=0.591). The only taxon significantly depleted with increasing Child–Pugh severity was *Edaphobaculum* (log FC=−0.47, *q*=0.032, AUC=0.659). Among these 21 taxa, 6 remained significant after in silico decontamination of biological samples using negative extraction controls (Figure 5C). Logistic regression analysis was performed for each of the 21 taxa, with 8 of them displaying a significant association with CP-C (*p*<0.05), after adjusting for gender and HCC development (Figure 5D). The strongest associations were observed for *OM27_clade* [AOR=2.81 (1.21–6.52), *p*=0.016], A0839 [AOR=2.32 (1.27–4.24), *p*=0.006]; Enterobacterales [AOR=2.25 (1.21–4.20), *p*=0.011]; Gaiellales [AOR=2.24 (1.21–4.16), *p*=0.011]; *Alteromonadaceae*/*Rheinheimera* [AOR=2.16 (1.16–4.03), *p*=0.015]; and Thermoleophilia [AOR=2.13 (1.17–3.88), *p*=0.013]. A negative association was observed for *Edaphobaculum* [AOR=0.28 (0.11–0.69), *p*=0.005].

## DISCUSSION

The increasing number of patients with MASLD-associated HCC, combined with the late diagnosis of this disease, warrants the need for earlier identification of those at risk for MASLD-associated HCC. Gut microbial signatures have shown promise as noninvasive biomarkers for the early detection of HCC.^21^,^22^,^23^ In this study, we aimed to determine whether bacterial DNA signatures in plasma can be used to predict HCC risk in MASLD patients with cirrhosis. To date, only a handful of circulating microbiome studies in HCC have been reported, all of which included patients of mixed etiologies.^30^,^31^,^35^ In addition, no such studies have been performed on longitudinal biospecimens from a prospective cohort of patients with cirrhosis under surveillance for HCC. The multicenter prospective cohort of patients with cirrhosis we developed is based on surveillance for HCC by contrast-enhanced MRI. This surveillance cohort, therefore, provides a unique opportunity to study biomarkers in blood prior to the diagnosis of early-stage disease. We previously reported several microbiome-related metabolites in serum, changing in abundance in the year prior to HCC diagnosis in patients with cirrhosis from this prospective cohort.^25^

The use of circulating bacterial genome studies is an attractive approach to biomarker discovery. An important limitation to this approach, however, is the susceptibility of low biomass samples to exogenous contamination introduced during the DNA extraction and sequencing processes.^28^ Therefore, negative experimental controls and decontamination frameworks are needed in order to detect genuine circulating bacterial DNA.^26^ Complete removal of all taxa previously recognized as putative contaminants may not be the optimal approach, as certain taxa can be both a contaminant and a true biological signal.^34^ The application of in silico decontamination strategies has been proposed, including the filtering of amplicon sequence variants with batch-dependent abundances and those with a higher prevalence in negative controls.^29^ In this study, taxa with greater abundance in the negative extraction controls than in the biological samples were excluded from all differential analyses. Furthermore, differential analyses were repeated after SCRuB decontamination,^34^ which partially removes putative contaminant reads based on batch-specific extraction controls. Finally, the identified differentially abundant genera were cross-referenced to a published list of 70 putative contaminants reported in negative controls by more than 1 study.^36^ Among all 14 genera identified as changed in our study, only 2 were on this list: *Acinetobacter* and *Staphylococcus*. Despite being putative contaminants, *Acinetobacter* and *Staphylococcus* species also represent prevalent human pathogens that typically occur in healthcare settings.^37^ In a large, multi-cohort population study of healthy humans that incorporated extensive removal of putative contaminants, both *Acinetobacter* and *Staphylococcus* species were among the top 30 microbes detected with high confidence in the circulation. Replication rate analysis also showed evidence of replication, suggesting the presence of viable microbial cells in the circulation rather than merely circulating DNA.^27^

The main finding of the nested cohort study was that bacterial genome signatures in plasma from patients with MASLD-associated cirrhosis were associated with HCC development, both by redundancy analysis and by PCoA. A separation in circulating bacterial DNA profiles between cirrhotic and HCC patients was previously observed in a cross-sectional study.^35^ Changes in patients with cirrhosis who developed HCC included an enrichment in *Tsukamurella*, *Bacteroides*, and *Staphylococcus* and a depletion of *Natronomonas*, with *Tsukamurella* and *Natronomonas* remaining significant after SCRuB decontamination. *Natronomonas* is a member of the Halobacteria class, which has been identified in the human gut.^38^ *Tsukamurella* has been found as a rare cause of opportunistic infections.^39^ Like the more well-known related *Mycobacterium*, *Tsukamurella* contains mycolic acids on its cell surface, which trigger immune responses and modulate cholesterol accumulation in cells.^40^ Overall, *Bacteroides* and *Staphylococcus* species were among the top 50 taxa detected in plasma microbial cfDNA from a large cohort of hospitalized patients.^41^ *Bacteroides* is one of the most abundant genera of the human gut, and was found to be enriched in the circulation of HCC patients, compared to MASLD patients^31^ or patients with cirrhosis.^30^ *Bacteroides* have been reported to also increase in patients with cirrhosis and severe portal hypertension.^32^ A step-wise increase in the relative abundance of *Staphylococcus* was observed in the serum of healthy controls, patients with cirrhosis, and HCC patients.^35^ Several HCC-associated taxa showed markedly improved prediction of HCC development when it occurred within 12 months prior to diagnosis, including Acidobacteriota, *Tsukamurella*, and *Staphylococcus*. An enrichment of Acidobacteriota in the gut was observed in patients with HBV-related cirrhosis and HCC.^42^ Acidobacteriota has also been detected in the liver tissue of MASLD patients^43^ and enriched in the tumor tissue of intrahepatic cholangiocarcinoma compared to adjacent non-tumoral liver tissue.^44^ Finally, we tested whether the performance of HCC-associated taxa in distinguishing between patients who developed HCC within 12 months and those who did not develop HCC could improve by adding information on the presence of LI-RADS-3 or LI-RADS-4 lesions. The LI-RADS classification system, initially released in 2011, allows for standardized characterization of liver lesions (pre-HCC and HCC) using CT or MRI with extracellular contrast agents.^45^ Remarkably, the combination of LI-RADS-3/4 lesion presence with HCC-associated taxa predicted HCC development within 12 months at an AUC of 0.864 (95% CI=0.795–0.932).

Other findings of the study were that bacterial genome signatures in plasma from patients with MASLD-associated cirrhosis were also associated with cirrhosis severity and gender, both by redundancy analysis and by PCoA. While male gender and increasing Child–Pugh severity commonly shared a significant association with 8 enriched taxa (7 unique) by differential analysis, HCC did not share any overlap in significant taxa with either gender or Child–Pugh class. Both male gender and Child–Pugh severity were associated with enrichment of the Proteobacteria phylum, Gammaproteobacteria class, and 2 lineages within this class: the Pseudomonadales order/*Moraxellaceae* family/*Acinetobacter* genus, and the Enterobacterales order/*Alteromonadaceae* family/*Rheinheimera* genus. Among the study patients, a significantly greater proportion of males presented with Child–Pugh classes B and C compared to females. Even after adjusting for both Child–Pugh class and HCC development, all 8 shared taxa remained significantly associated with male gender. On the other hand, only Enterobacterales, *Alteromonadaceae*, and *Rheinheimera* were significantly associated with CP-C when adjusting for gender and HCC development. After SCRuB decontamination, *Alteromonadaceae* and *Rheinheimera* remained significantly associated with male gender, while Gammaproteobacteria and Enterobacterales remained significantly associated with Child–Pugh severity. Gammaproteobacteria have been found to be enriched in the gut of patients with liver cirrhosis,^46^ while *Moraxellaceae* and *Acinetobacter* have been detected in ascitic fluid from patients with liver cirrhosis.^47^ A step-wise enrichment of Gammaproteobacteria abundance from normal liver tissue, to peritumoral HCC tissue, to primary HCC tissue has also been reported.^48^ While the Enterobacterales order contains multiple pathogenic bacteria, including *Escherichia* and *Shigella* species, the majority of reads in our study were assigned to *Rheinheimera*. *Rheinheimera* has been detected in several human microbiome niches as well as in blood.^49^ Additional changes observed with increasing Child–Pugh class included an enrichment in Gaiellales and its members *Gaiellaceae* and *Gaiella*; *OM27_clade*, and *A0839*. *Gaiella* has been detected in the gastric mucosa of patients with gastric cancer, where its abundance was negatively correlated with Foxp3^+^ regulatory T cells, suggesting immunostimulatory properties.^50^ The *A0839* family has been detected in ascitic fluid from patients with liver cirrhosis.^47^

This study has several limitations. Although several strategies were implemented to reduce the influence of low biomass contamination, there is a lack of consensus on the optimal approach. Therefore, contamination may still exert an influence on the results. Secondly, while the samples are from a multicenter cohort, validation with an independent prospective cohort will be required to demonstrate both reproducibility and broad applicability. In particular, the predominantly non-Hispanic white demographic homogeneity of the study cohort may limit the applicability of the reported associations to other populations.

In conclusion, we identified bacterial taxa in the circulation of patients with MASLD-associated cirrhosis that were associated with gender, Child–Pugh class, and HCC development, many of which are known to be human-associated. Whether the taxa we identified act as bystanders of dysbiosis or as key players in Child–Pugh severity or HCC development remain to be determined. The origin of the identified circulating bacterial cfDNA is also unknown. The identified circulating bacterial DNA signatures may have utility in personalized approaches to HCC surveillance in MASLD patients.

## Supplementary Material

## Data Availability

The 16S rRNA sequencing data have been deposited into the Sequence Read Archive (SRA) of the National Center for Biotechnology Information (NCBI) with BioProject accession number PRJNA1186240. Conceptualization: Laura Beretta and Suet-Ying Kwan; methodology: Suet-Ying Kwan, Tiffany L. Calderone, Lillian I. Dolapchiev, Prasun K. Jalal, David W. Victor, and Laura Beretta; formal analysis: Suet-Ying Kwan, Lillian I. Dolapchiev, and Tiffany L. Calderone; investigation and resources: Lillian I. Dolapchiev, Jessica I. Sanchez, Caren I. Sanchez, Tiffany L. Calderone, Megha B. Bhongade, Ahmed El Sabagh, David W. Victor, Nakul Gupta, Prasun K. Jalal, and David W. Victor; writing—original draft: Lillian I. Dolapchiev, Suet-Ying Kwan, and Laura Beretta; visualization: Suet-Ying Kwan and Lillian I. Dolapchiev; supervision: Laura Beretta, Tiffany L. Calderone, Prasun K. Jalal, and David W. Victor; project administration: Tiffany L. Calderone; funding acquisition: Laura Beretta. This study was supported by NIH/NCI R01 CA195524 to Laura Beretta.
