# Supplementary material for: Blood bacterial DNA signatures in a prospective cohort of patients with MASLD cirrhosis
Source: Hepatol Commun. 2025 Jun 9;9(7):e0722. doi: 10.1097/HC9.0000000000000722 (PMC12150986; doi:10.1097/HC9.0000000000000722)
Supplement: Supplementary file 1 [file hc9-9-e0722-s001.docx]

**Supplemental Table S1. Demographic and clinical parameters of the 151 MASLD patients with cirrhosis included in the study.** Data are displayed as n (%) or mean (range) - median. ALP: alkaline phosphatase; ALT: alanine aminotransferase; AST: aspartate aminotransferase; MELD score: model for end stage liver disease; Dx: diagnosis.

| **Demographics** | **ALL (n=151)** |
| --- | --- |
| **Gender (male)** | 63 (41.7%) |
| **Race** |  |
| *White* | 145 (96.0%) |
| *Black* | 1 (0.7%) |
| *Other* | 5 (3.3%) |
| **Ethnicity** |  |
| *Hispanic* | 41 (27.2%) |
| *Non-Hispanic* | 110 (72.8%) |
| **Co-Etiology** |  |
| *Alcohol* | 15 (9.9%) |
| **Ascites** |  |
| *Absent* | 58 (38.4%) |
| *Controlled medically* | 83 (55.0%) |
| *Poorly controlled* | 10 (6.6%) |
| **Encephalopathy** |  |
| *Absent* | 63 (41.7%) |
| *Controlled medically* | 81 (53.6%) |
| *Poorly controlled* | 7 (4.6%) |
| **Child Pugh Class** |  |
| *Child Class A* | 57 (37.7%) |
| *Child Class B* | 74 (49.0%) |
| *Child Class C* | 20 (13.2%) |
| **Child Pugh Score** | 7 (5-13)-7 |
| **Diabetes** | 104 (68.9%) |
| **Age** | 62 (30-81) - 63 |
| **Body Mass Index** | 35.1 (19.0-58.3) - 33.8 |
| **MELD Score** | 12 (6-23) - 11 |
| **HCC Dx** | 25 (16.5%) |
| **Clinical Labs** |  |
| **Protein (g/dL)** | 7.2 (5.3-9.3) - 7.2 |
| **Albumin (g/dL)** | 3.5 (1.9-5.0) - 3.5 |
| **Total Bilirubin (mg/dL)** | 1.7 (0.2-9.5) - 1.3 |
| **Bilirubin Direct (mg/dL)** | 0.6 (0.1-4.4) - 0.5 |
| **ALK (U/L)** | 135.1 (11-435) - 118 |
| **AST (U/L)** | 46.4 (15-239) - 41 |
| **ALT (U/L)** | 33.0 (10-107) - 29 |
| **Platelets (billion/L)** | 112.6 (23-345) - 100 |
