# Supplementary material for: Blood bacterial DNA signatures in a prospective cohort of patients with MASLD cirrhosis
Source: Hepatol Commun. 2025 Jun 9;9(7):e0722. doi: 10.1097/HC9.0000000000000722 (PMC12150986; doi:10.1097/HC9.0000000000000722)
Supplement: Supplementary file 2 [file hc9-9-e0722-s002.docx]

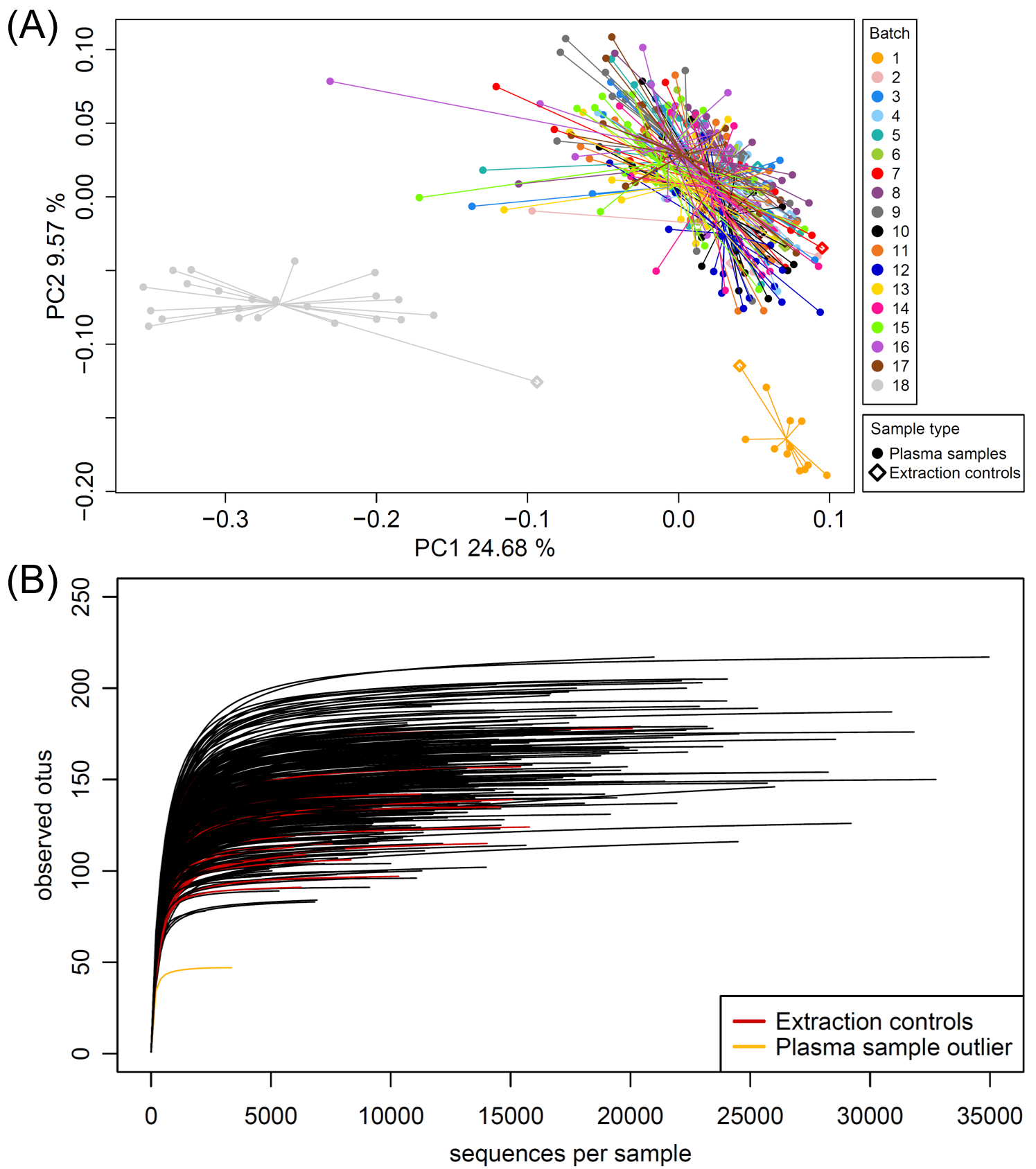


**Supplemental Figure S1.** (A) PCoA plot of extraction controls and patient cfDNA samples based on weighted UniFrac distances, with samples grouped by extraction batch. (B) Rarefaction curves showing number of OTUs observed in each extraction control and patient cfDNA sample by sequencing depth.


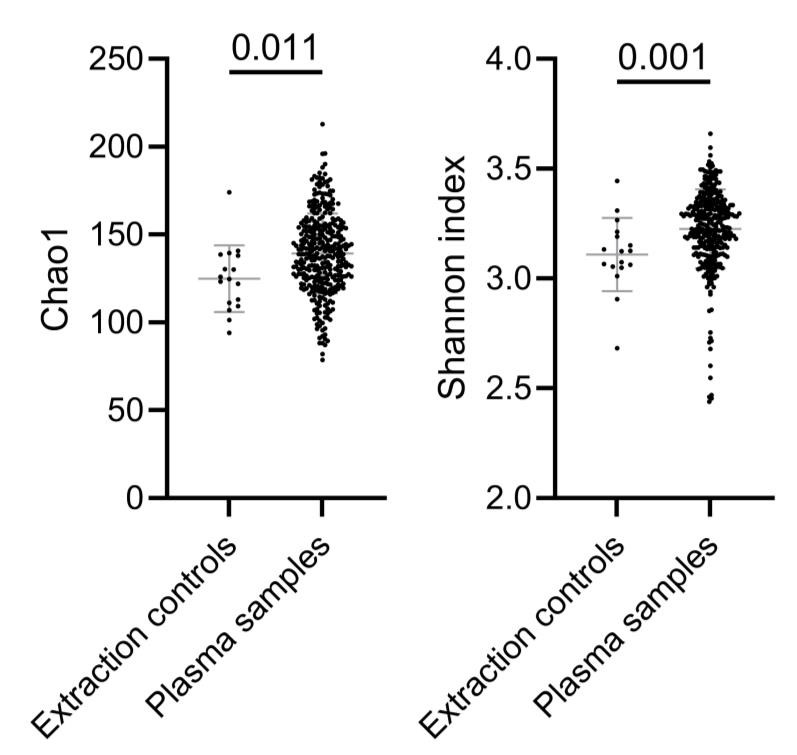


**Supplemental Figure S2.** Alpha diversity of extraction controls versus plasma cfDNA samples. Alpha diversity metrics for richness (Chao1) and richness and evenness (Shannon index) were calculated on count data rarefied to 2253 reads.
